# Barriers to and incentives for achieving partograph use in obstetric practice in low- and middle-income countries: a systematic review

**DOI:** 10.1186/1471-2393-14-281

**Published:** 2014-08-16

**Authors:** Elizabeth Ollerhead, David Osrin

**Affiliations:** UCL Institute for Global Health, 30 Guilford Street, London, WC1N 1EH UK

**Keywords:** Obstetrics, Labor, Obstetric, Health knowledge, Attitudes, Practice, Quality of health care, Developing countries, Review, Systematic

## Abstract

**Background:**

The partograph is a graphic display of the progress of labour, recommended by the World Health Organization, but often underused in practice in low- and middle-income countries. We were interested in going beyond demonstration of potential efficacy – on which the existing literature concentrates - through a systematic review to identify barriers to and incentives for achieving partograph use.

**Methods:**

We searched Ovid MEDLINE, Ovid Maternity and Infant Care, POPLINE, Web of Science, and Scopus, from 1^st^ January 1994 to 30^th^ September 2013, using the term ‘partogra*’ to include ‘partograph’, ‘partogram’, or ‘partogramme’. The selection criteria were for primary or secondary research describing barriers to and incentives for partograph use in low- and middle-income countries, in English, reported in peer-reviewed publications since 1994. Thematic analysis of text on partograph use was applied to a commonly used framework for change in clinical practice, with levels describing the innovation, the individual professional, the woman, and social, organisational, economic and political contexts.

**Results:**

Reported barriers to and incentives for partograph use related to the partograph itself, professional skills and practice, clinical leadership and quality assurance, and the organisational environment within the wider provision of obstetric care. Neither the evidence base for its effectiveness, nor its credibility, was reported as a barrier to use.

**Conclusion:**

Identifying and addressing local barriers and incentives in low- and middle-income countries, based on those in published research, could inform strategies to improve partograph use. Emerging technologies could be used to address some barriers. The thresholds for essential maternity care at which the partograph adds value should be further evaluated.

## Background

A graphic display of the progress of labour, the partograph helps skilled birth attendants to recognise emerging difficulties and take action according to a clinical management protocol. Partography can reduce the burden of obstructed labour as a cause of maternal and fetal death, obstetric fistula, and fetal hypoxia, birth trauma and infection [1]. Requiring only a printed sheet to chart what should be routine care, the partograph would seem to be an appropriate technology with a clear place in maternity care in low- and middle-income countries (LMICs). It is variably used, however, and often reported as substantially underused [2].

One response to this shortfall is to demonstrate potential efficacy and effectiveness. The WHO prospective trial in South East Asia suggested that partography with a management protocol reduced prolonged labour and promoted more appropriate obstetric assessment and intervention [3], although a Cochrane review of trials in variably resourced settings found insufficient evidence to either support or discourage its use [4]. Some gaps remain in the evidence base, and the assumption of universal relevance in a diversity of contexts and for a diversity of women is still debated [5]. Our concern is less that the partograph might have failed tests of efficacy, and more that it has been lost in translation. Wide variation in use between countries has been reported, even in comparisons at similar levels of health systems [2], suggesting that contextual factors may be important influences. “One of the most consistent findings in health services research is the gap between best practice (as determined by scientific evidence), on the one hand, and actual clinical care, on the other” [6]. Because we wanted to understand the literature on usage as a first step to addressing practice [7], we did a systematic review of barriers to and incentives for partography in LMICs. If we assume that the partograph is a technology with an evidence-based role in obstetric care, its adoption, regular use, and quality fall into the realm of change in practice. Healthcare workers are well aware of the complexities inherent in changing practices, and implementation theory is increasingly contributing to managing change [8, 9]. In framing the findings of our review, we used a classification suggested by Grol and Wensing [6] that has pedigree in analyses of healthcare by, among others, the UK National Institute for Health and Clinical Excellence [10] and the Australian National Institute of Clinical Studies [11].

## Methods

The study was developed as a dissertation based on an iterative process rather than a protocol. Journal publications about partography, in English, were identified from 1994, when the composite WHO partograph was recommended. We selected 1994 as the starting point because it was a clear policy moment following the updated WHO guidance. After partograph development reported by Philpott [12], early studies researched efficacy, application to different populations, and clinical scenarios and outcomes. Most publications related to high-income countries, and descriptions of issues around use in practice in LMICs became a more common theme around 2000.

We searched Ovid MEDLINE, Ovid Maternity and Infant Care, POPLINE, Web of Science, and Scopus, from 1st January 1994 to 30th September 2013, using the term ‘partogra*’ to include the words ‘partograph(s)’ , ‘partogram(s)’ or ‘partogramme(s)’ (1 partogra*.mp. [mp = title, abstract, original title, name of substance word, subject heading word, keyword heading word, protocol supplementary concept, rare disease supplementary concept, unique identifier. 2 limit 1 to (english language and yr = "1994 -Current")]). Search results were imported into EndNote X5. Primary or secondary research was selected, referring to LMICs using the 2012 World Bank Classification. Publications that were not research or reviews – including reports, books, news articles, editorials, and letters - were excluded due to limited detail on partograph use. We chose not to include theses and conference papers. Publications reporting reasons for partograph use and non-use were included in the synthesis, while those that only quantified use were excluded. We copied text about barriers and incentives to partograph use into a table and rearranged it into provisional themes, which identified that reported issues for use were predominantly about individual professionals in the context of their working environment, rather than concerns about the evidence-base for partographs. The provisional themes were reconciled with the practice change framework [6]. Table 1 outlines the thematic classification of barriers to and incentives for partograph use, based on the framework by Grol and Wensing [6]. The identification, selection and analysis were carried out by one reviewer (EO). Although bias presumably exists in the sense that publications were more likely to arise from situations in which the introduction of partography had been unsuccessful, the qualitative nature of the data meant that we were unable to quantify risk.

**Table 1:** **Classification of barriers to and incentives for partograph use in low and middle income countries**

Theme [6]	Characteristics [6]	Incentives identified in thematic analysis	Barriers identified in thematic analysis
The innovation itself	Advantages in practice	Monitoring labour	Availability
	Feasibility	Continuity of care	Graphing skills
	Credibility		Language
	Accessibility		Literacy
	Attractiveness		Many partograph versions
Individual professional	Awareness	Skilled birth attendants	Awareness
	Knowledge	Positive attitude	Knowledge
	Attitude		Skills
	Motivation to change		Commitment
	Behavioural routines		Confidence
			Negative attitude
Patient (Woman)	Knowledge	Knowledge	Late admission
	Skills		
	Attitude		
	Compliance		
Social context	Opinion of colleagues	Leadership	Inter-professional barriers
	Culture of the network	Staff involvement	Lack of:
	Collaboration	Supervision	- Engagement
	Leadership	Monitoring	- Leadership
		Audit	- Role models
		Evaluation	- Facilitation
			- Monitoring
			- Evidence-based practice
			Retrospective documentation
Organisational context	Organisation of care processes	Supporting policy	Lack of guidelines
	Staff	Staff involvement	Poor record keeping
	Capacities	Teamwork	Shortage of equipment, clinical supplies,
	Resources		and medicines
	Structures		Workload
			Understaffing
			Frequent staff rotation
			Demoralised staff
Economic and political context	Financial arrangements	National policy	Deficiencies in service provision for maternity care
	Regulations	Medico-legal duty	
	Policies		

### Role of the funding source

The sponsor had no role in the study design, data collection, analysis, interpretation or writing of the article. EO had access to all study data and responsibility for the decision to submit for publication.

## Results

**Figure 1:**
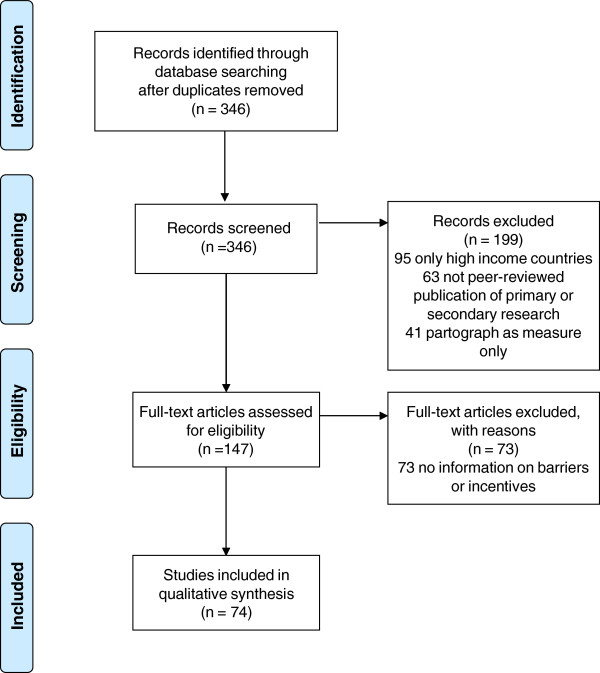
**Flow diagram for study selection.**

There were 346 references after removal of duplicates. All 346 abstracts were screened to select publications in which partograph use was described in practice. 199 papers were excluded at screening – 95 only related to high-income countries, 63 were not primary or secondary research in peer-reviewed publications, and 41 only mentioned the partograph as a measure for testing other clinical interventions. 147 publications were selected for full review. 73 were excluded because they provided no information on barriers or incentives for use. The sequence of review is summarised in the PRISMA [13] Flow Diagram in Figure 1.

There were 74 publications for narrative synthesis on partograph use: 64 were studies of healthcare, including 18 audits, 18 surveys, 20 practice assessments, and 8 training evaluations; 10 were reviews. Of 59 primary studies from a single country, 43 were from Sub-Saharan Africa, 9 from South Asia, and the rest from other Asian countries.

### Innovation

The innovation may be a barrier or an incentive, depending on its perceived advantages in terms of practice, feasibility, credibility, accessibility, and attractiveness [6]. The main advantage of the partograph was that it was seen as useful for monitoring labour and continuity of practice [14, 15]. Neither the evidence base for its effectiveness, nor its credibility, was reported as a barrier to use. Partography was, however, perceived as time-consuming [16–20]. Differences between the available versions could be confusing [21], and their completion required training. Difficulties tended to arise when birth attendants’ graphing skills were less developed than their obstetric knowledge [22, 23]. Identifying the latent phase of labour was a particular area of confusion [16, 21, 24–26], and it is possible that the simplified version of the partograph may be more likely to be completed [27]. The requirement for a certain level of literacy [28], and for translation into local languages [18] were potential barriers to uptake. Finally, you cannot use a partograph if you do not have it: procurement and supply chains are often problematic in LMICs, and limited availability was reported [1, 15, 19, 29–35].

### Individual professional

For individual practitioners, barriers to and incentives for change relate to their awareness, knowledge, attitude, motivation to change, and behavioural routines [6]. Effective partograph use requires health workers to “internalise its function, namely continuous monitoring, documentation and interpretation of collected information leading to early detection and prevention of neonatal and maternal complications” [16]. Where this occurred, professionals valued the partograph highly [14, 36]. The literature included examples of instances in which this had not occurred and the partograph was not valued by staff [18], with a lack of “buy in” [37, 38]. Low awareness of the partograph itself was an issue [17, 32, 34], and lack of knowledge was widely reported, often within a wider deficit of knowledge and skills in maternity care [1, 15, 16, 18, 19, 32, 34, 35, 39–46]. Other barriers included attitude and limited confidence [15, 18], variation in commitment [34], and inadequate interaction with women [47].

### Woman

The knowledge, skills, attitude and compliance of the woman are possible barriers to or incentives for change [6], though these were not reported relating to partograph use in LMICs, where user empowerment can be low. Training during pregnancy for women and their families to recognise prolonged labour is part of fistula prevention programmes [48, 49], with the potential to promote partograph use. An important factor reported as limiting partograph use was admission late in labour [26, 38, 47, 50–52]. Clearly, some women who present later in labour may be progressing well, but others may be experiencing complications that could have been be prevented by earlier partograph monitoring*.*

### Social context

The opinions of colleagues, organizational culture, collaboration and leadership are barriers or incentives in the social context of clinical practice [6]. Supportive culture [53], including senior leadership [16, 35, 54], and staff involvement [37, 55, 56], promotes partograph use. Despite training, student midwives found partography difficult without role models and leadership from senior staff, along with facilitation in the clinical setting [38, 57]. Senior midwives themselves might find the idea of learning from junior colleagues uncomfortable [44], and the social and professional barriers between midwives, physicians and managers, could also be obstacles to the process of implementation, particularly if combined with a sense of professional inadequacy [47]. Once established, supervision and monitoring [1, 15, 42, 58], and audit and evaluation [1, 16, 56, 59, 60], were important to the maintenance of partograph use and quality. Without them, some social contexts allowed poor practice such as the acceptance of retrospective documentation, with completion of partographs after delivery and before discharge particularly well-described [16, 35, 38, 47, 50, 57]. Contexts lacking a culture of evidence-based practice [47], or monitoring [50, 61, 62], were also barriers to partograph use.

### Organisational context

Organisation of care processes, human resources, capacities, resources and structures raises both barriers and incentives [6]. Supportive organisational policy promoted partograph use [1, 61], but introduction without guidelines reduced it [15]. Valuing partography as a tool for teamwork that matched provider skills with the needs of women could benefit practice [63], and involving staff in implementation was helpful [55, 64]. Care processes such as the requirement for duplicate recording in notes and partographs reduce completion [1], but low use might also represent a wider deficit in record-keeping [35, 59]. The most commonly reported organisational barriers were understaffing and high workload [1, 15, 19, 32, 35, 44, 61, 62, 65]. Frequent staff rotation [47], and job dissatisfaction also limited use [65]. Key barriers related to broader deficiencies in obstetric care [65], including shortage of equipment for measurement [28], oxytocin for labour augmentation [17], and other clinical equipment and supplies [44, 50, 53].

### Economic and political context

Clinical care is a reflection of the broader economic and political environment. Barriers or incentives include financial arrangements, regulations and policies [6]. Supportive national policy assisted partograph use [62], and its requirement as a medico-legal duty could emphasise its value [61]. The same duty of completion could, however, lead to recording of false data and a missed opportunity to improve clinical care [38]. Studies referred to the wider deficit in comprehensive systems for obstetric care and the WHO study noted underlying “geographical, economic, political and sociocultural” constraints [66].

## Discussion

### Main findings

Partography is well established in many settings, and the synthesis aimed to help us consolidate its use. Many reported barriers to and incentives for partograph use affected local practice in LMICs. These related to the professional and practice environment of obstetric care, rather than to the evidence base. Partographs needed to be available, with appropriate equipment and clinical supplies for assessing progress in labour, and the resources to provide recommended interventions. Professionals might lack awareness, knowledge and training, and under-value partographs, seeing completion as complex and time-consuming rather than assisting good practice. A supportive professional environment from peers and leaders, with quality assurance systems, promoted partograph use. Adaptation to the local context was often needed in terms of both language and clinical practice. Empowerment of women to expect better care, with delivery at health facilities and earlier admission, would be likely to increase future partograph use.

### Strengths and limitations

Ours was a comprehensive review of partograph use in LMICs, based on reports of practice published in journals. The exclusion of non-English papers resulted in low representation of experience from francophone Africa and Latin America, and there may also have been material in the grey literature that was not examined. However, it is likely that many of the barriers and incentives would be common to health facilities in different places, and the focus on local assessment could identify relevant factors.

### Interpretation

A multilevel approach to assessing barriers to and incentives for change [6] can provide a framework to improve partograph implementation and use. Local barriers and incentives from this review could be assessed by seeking information from healthcare workers, or observing clinical practice, and matched to methods for changing practice [10]. Training is most frequently reported as a tool for better practice and lack of skills and knowledge are common. While educational initiatives address this, other approaches to changing behaviour, focused on cognition, attitude and motivation, may be important for the individual professional [6]. Shaping the social network and developing leadership, with assessment and accountability for clinical quality improvement, could support the application of learning. Wider organisational opinion needs to be supportive of partography, but deficiencies in staffing, supplies and maternity care, both limit partograph use, and reduce the likelihood of evidence-based practice.

Further consideration of new technologies for the partograph may address some barriers and support use [1]. For example, electronic partographs - into which raw data are entered and internally formatted and charted - could overcome graphing difficulties. Linking electronic partography with a management protocol could prompt clinical assessment and action, overcoming gaps in staff knowledge. The PartoPen, a digital pen partograph, is another technology being tested to assist data validation and provide clinical prompts and decision support [67]. The need for training, supervision and follow-up remains, but future use of e-learning could increase knowledge [23]. New opportunities for communication, education and decision support through mobile devices (mHealth) could also provide maternity care workers with remote advice and produce data for feedback and audit [68]. These technologies and other adaptations of the partograph, in order “to be of optimal value to the users” [14] in different settings, could facilitate use.

Partographs can only improve outcomes within an effective maternity care system. Essentials for their use are skilled birth attendants working with a labour management protocol, with appropriate training, supervision and monitoring for quality assurance, and supportive policy [1]. Deficiencies in obstetric care and health systems were recurrent themes in this review. Further evaluation of the place of the partograph in maternity care should shape strategy as services develop in low resource settings, using the body of evidence on partograph use in LMICs to inform appropriate and optimal use in different settings with varying resources, and to identify remaining evidence gaps for future research.

## Conclusion

Partographs are often underused in low- and middle-income countries. Reported barriers to and incentives for use have been reviewed as a basis for local assessment, relating to the innovation, woman, individual professional, and social, organisational and economic and political contexts. Identifying local barriers and incentives should inform strategies to improve use. Emerging technologies to support electronic partograph recording, with clinical prompts and remote decision support, may also address some barriers. The thresholds for essential maternity care at which the partograph adds value should be examined as services develop in low resource settings.

## Authors’ information

EO is a consultant in public health. DO is a paediatrician and public health researcher, Wellcome Trust Senior Research Fellow in Clinical Science, with a particular interest in women’s and children’s health in low and middle income countries.

## Abbreviations

LMICs: Low and middle income countries, as defined by the World Bank 2012
WHO: World Health Organization.
